# Mobile Gesundheitstechnologien, soziale Gerechtigkeit und populationsbezogene Vulnerabilitäten: Eine Public-Health-ethische Perspektive auf mHealth am Beispiel von Diabetes mellitus Typ 2

**DOI:** 10.1007/s00103-022-03650-8

**Published:** 2023-01-25

**Authors:** Bianca Jansky, Felix Machleid, Verina Wild

**Affiliations:** 1 Medizinische Fakultät, Ethik der Medizin, Universität Augsburg, Universitätsstr. 2, 86159 Augsburg, Deutschland; 2 Institut für Soziologie, Ludwig-Maximilians-Universität, München, Deutschland; 3 Charité-Universitätsmedizin Berlin, corporate member of Freie Universität Berlin, Humboldt-Universität zu Berlin, Berlin, Deutschland; 4 Berlin Institute of Health at Charité – Universitätsmedizin Berlin, Berlin, Deutschland

**Keywords:** Gesundheitsapp, Gesundheitsgerechtigkeit, Diabetes mellitus Typ 2, Public-Health-Ethik, Eigenverantwortung, health app, Health equity, Type 2 diabetes mellitus, Public health ethics, Individual responsibility for health

## Abstract

Mobile Gesundheitstechnologien (mHealth) fördern den Trend hin zu Eigenverantwortung und Selbstmanagement. Ziel des Beitrags ist es, am Beispiel von Diabetes mellitus Typ 2 (T2DM) die Diskussion zu mHealth, Eigenverantwortung und Gerechtigkeit – welche es bisher nur in Ansätzen gibt – aus einer Public-Health-ethischen Perspektive zu vertiefen. Dabei zeigt sich, dass mHealth im Bereich T2DM soziale Gesundheitsgerechtigkeit einerseits verbessern, andererseits aber auch soziale Gesundheitsungerechtigkeiten verschärfen kann. Aus einer gerechtigkeitsfokussierten, Public-Health-ethischen Perspektive auf T2DM-mHealth ist es notwendig, besser zu verstehen, ob und wie vulnerable Bevölkerungsgruppen bei mHealth-Entwicklung und -Einsatz mitbedacht werden, wie sie die Nutzung der Technologie erleben, welche sozialepidemiologischen Auswirkungen der zunehmende Einsatz von mHealth haben kann, welche gesundheitlichen Ungleichheiten im Bereich T2DM ungerecht sind, inwieweit die Eigenverantwortung in die Hände der Nutzenden gelegt werden soll und wo die Grenzen der Eigenverantwortung liegen. Die Berücksichtigung der sozialen Diversität und der sozialen Determinanten von Gesundheit ist ein stetiger Prozess und muss alle Phasen der Entwicklung und des Einsatzes von mHealth durchziehen.

## Abstract (en)

Mobile health technologies (mHealth) promote the trend towards personal responsibility and self-management. By using the example of type 2 diabetes mellitus (T2DM), the article aims to deepen the discussion on mHealth, personal responsibility and justice—which has so far only been rudimentary—from a public health ethical perspective. It shows that in the field of T2DM, mHealth can on the one hand improve social health justice, but on the other hand can also exacerbate social health *injustices*. From a justice-focused, public health ethical perspective on T2DM mHealth, it is necessary to better understand whether and how vulnerable population groups are considered in mHealth development and implementation, how these groups experience the use of the technology, what social–epidemiological impacts the increasing use of mHealth can have, which health inequalities in the area of T2DM are unfair, to what extent personal responsibility should be placed in the hands of the users, and where the limits of personal responsibility lie. Considering social diversity and the social determinants of health is an ongoing process and must permeate all phases of mHealth development and implementation.

## Hintergrund

Der Stellenwert von Krankheitsmanagement, Gesundheitsförderung und Prävention im Bereich von Diabetes mellitus Typ 2 (T2DM) ist groß. Individuelles Verhalten und Eigenverantwortung für Gesundheit sind hier entscheidende Elemente, bei denen mobile Gesundheitstechnologien (im Folgenden „mHealth-Technologien“ oder „mHealth“) helfen können, indem sie die Gesundheitsprävention und -förderung sowie das Krankheitsmanagement verstärkt in die Hände der Nutzenden legen.

Der Begriff „mHealth“ ist nicht einheitlich definiert und wird oft als eine Unterkategorie von „eHealth“ verstanden, welches alle elektronischen und digitalen Gesundheitstechnologien umfasst. Spezifisch für mHealth ist, dass die Anwendungen über Smartphones, digitale Assistenten und andere intelligente Geräte für medizinische und öffentliche Gesundheitszwecke *mobil* bereitgestellt werden, z. B. als Apps [1]. mHealth wird vielfältig eingesetzt, z. B. für Prävention, Diagnostik, Therapie, Nachsorge, Patient*innenmonitoring und für administrative Zwecke. Bei T2DM können Apps u. a. als Tagebücher, personalisierte Erinnerungshilfen oder als Motivations- und Trainingshilfen eingesetzt werden. Für T2DM gab es bereits 2013 die meiste wissenschaftliche Literatur zu mHealth [2].

Mit mHealth sind große Hoffnungen verbunden, auch zur Verringerung gesundheitlicher Ungleichheiten [3]. Diese Hoffnungen richten sich u. a. auf das *Empowerment*, also eine Erleichterung der eigenverantwortlichen Partizipation von Patient*innen an ihrer Gesundheitsprävention und -förderung, und auf einen niedrigschwelligen Zugang zu Gesundheitsdienstleistungen durch digitale Lösungen. Im Jahr 2021 besaßen 62 Mio. Personen in Deutschland ein Smartphone, Tendenz steigend [4]. Marktforschungsdaten zeigen, dass 75 % der Smartphone-Besitzer*innen eine Gesundheits-App nutzen [5]. Aktuell (2022) nutzen 275.000 Personen eine Diabetes-App, ebenfalls mit steigender Tendenz [6].

Der Einsatz von Gesundheits-Apps wird in Deutschland auch von gesundheitspolitischer Seite gefördert. Seit 2020 sind Digitale Gesundheitsanwendungen (DiGA) Teil der Regelversorgung und können nach ärztlicher Verordnung durch Krankenkassen erstattet werden [7]^1^.

Die ethischen Aspekte von mHealth wurden früh thematisiert, und es liegen entsprechende Rahmenwerke und Richtlinien vor [8–10]. Diskutiert werden auch Aspekte der Gerechtigkeit („health equity“), wobei häufig ein gleichberechtigter Zugang zu den Technologien sowie „digital literacy“, also die Kompetenz im Umgang mit diesen Technologien, gefordert wird. Zudem liegen soziologische Arbeiten vor zur Notwendigkeit, mHealth und Eigenverantwortung für Gesundheit vor dem Hintergrund ungleicher sozialer Bedingungen und dem politischen und wirtschaftlichen Kontext zu diskutieren [11]. Eine vertiefte Diskussion zu Eigenverantwortung für Gesundheit, sozialer Gerechtigkeit und populationsbezogenen Vulnerabilitäten, wie sie in der Public-Health-Ethik in Bezug auf andere Themen kenntnisreich geführt wird, beginnt bisher jedoch erst in Ansätzen [12–16]. Die spezifische ethische Diskussion von Gerechtigkeitsaspekten zu mHealth im Bereich T2DM ist dadurch erschwert, dass nur spärliche empirische Ergebnisse zu sozialen Ungleichheiten in der Nutzung in diesem Bereich vorliegen. Allerdings ist gerade die Prävention und das Management von T2DM prädestiniert für eine breite Nutzung von mHealth, und gleichzeitig ist diese weit verbreitete Erkrankung durch einen sozialen Gradienten gekennzeichnet. Es ist daher als Anwendungsbeispiel gut geeignet, aus dem sich Überlegungen auch für andere Bereiche ergeben können.

Im vorliegenden Beitrag gehen wir zunächst darauf ein, wie mHealth zum Selbstmanagement bei T2DM eingesetzt wird und wie hierbei die Eigenverantwortung für Gesundheit gefördert werden soll. Nachfolgend fassen wir Grundlagen zu sozialen Determinanten für Gesundheit und soziale Gerechtigkeit zusammen. Anschließend führen wir die Erkenntnisse zusammen, diskutieren den Einfluss von mHealth im Bereich T2DM auf soziale Gesundheitsungerechtigkeiten und schließen mit einem Fazit.

## Selbstmanagement bei T2DM und mHealth-Technologien

**Figure 1:**
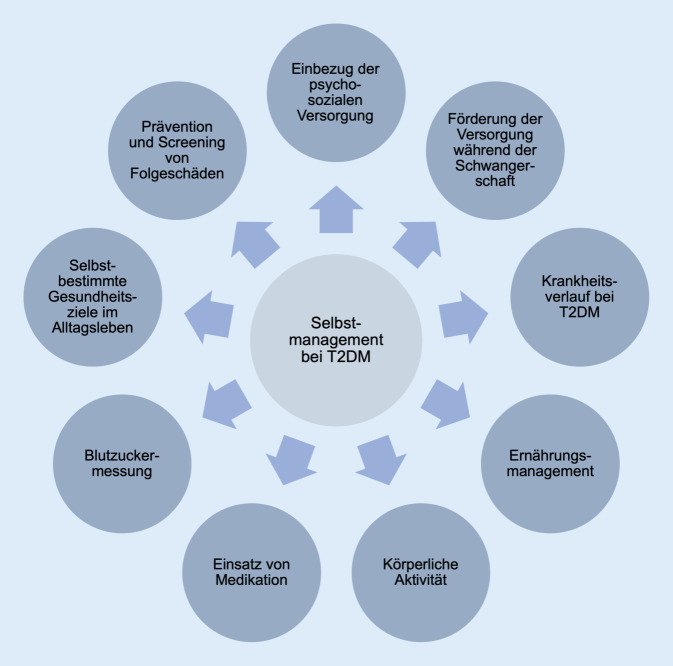

Die Stoffwechselerkrankung T2DM tritt aufgrund einer Insulinresistenz auf, die meist auf zu wenig Bewegung, Adipositas oder höheres Alter zurückzuführen ist. Während die Prävalenz weltweit erheblich zunimmt, liegt sie in Deutschland seit 1998 bei rund 9 % [17]. Für einen günstigeren Krankheitsverlauf ist ein ausgeprägtes Selbstmanagement entscheidend – u. a. in den Bereichen Ernährung, körperliche Aktivität, Blutzuckermessung, Einsatz von Medikation (Abb. 1; [18, 19]). Dies setzt ein hohes Maß an Motivation und Gesundheitskompetenz voraus [20]. Auch bevor mHealth zum Selbstmanagement bei T2DM eingesetzt wurde, spielte Eigenverantwortung in Form von analogen Tagebüchern und Blutzuckerprotokollen eine wichtige Rolle.

Seit 2015 hat sich die Anzahl derjenigen, die diabetesspezifische mHealth-Technologien nutzen, ungefähr vervierfacht [21]. Studien bestätigen, dass mHealth Lebensstilveränderungen bei T2DM unterstützt, zur Senkung des Hämoglobin-A1c-Werts (HbA1c) beiträgt, sich positiv auf diabetesbezogenen Distress, Hypoglykämieangst und gesundheitsbezogene Lebensqualität auswirkt und die Anzahl von Notaufnahmebesuchen und Krankenhausaufenthalten verringern kann [22–25]. Das größte Potenzial in der Patient*innenversorgung wird dabei in Disease-Management-Apps gesehen [26]. In Deutschland sind diabetesbezogene mHealth-Technologien mittlerweile so etabliert, dass die Deutsche Diabetes Gesellschaft mit der Initiative „DiaDigital“ eine Qualitätsevaluation anbietet (https://www.diabetesde.org/diadigital). Eine solche Qualitätsüberprüfung gibt jedoch nicht unbedingt Aufschluss über die Nutzer*innenfreundlichkeit oder ethische Fragen [27].

Während es eine Vielzahl an T2DM-spezifischen mHealth-Technologien gibt, wurde gezeigt, dass Betroffene auch andere Anwendungen (generelle Lifestyle- und Gesundheitsmessaging-Apps, Websites mit Gesundheitsinformationen, Foren) aus dem gesamten mHealth-Spektrum nutzen und so ihr Krankheitsmanagement personalisieren [28].

## Soziale Determinanten von Gesundheit, sozialer Gradient und (Grenzen der) Eigenverantwortung für Gesundheit

Gesundheit und Krankheitslast sind innerhalb der Bevölkerung ungleich verteilt. Dabei spielen soziale Ungleichheit sowie Möglichkeiten der Verhaltens- und Verhältnisprävention eine große Rolle [29].

Strukturelle Ungleichheiten sowie der soziale Gradient bei Erkrankungen sind in den letzten Jahrzehnten empirisch herausgearbeitet worden [29–32]. Auch T2DM unterliegt einem sozialen Gradienten, der bedingt ist durch komplexe biopsychosoziale Zusammenhänge. Bei benachteiligtem sozialem Status sind Prävalenz der Risikofaktoren, Inzidenz und Prävalenz von T2DM sowie Komplikationen erhöht [33]. Das bedeutet, dass es unter sozial benachteiligten Gruppen mit T2DM im Vergleich zu besser gestellten Bevölkerungsgruppen höhere Raten an unzureichender Blutzuckereinstellung, vermehrter Hyperglykämie, diabetesbedingten Krankenhausaufenthalten und Komplikationen sowie vorzeitiger Mortalität und Folgeschäden gibt [24, 34].

Wenn soziale Determinanten und ein sozialer Gradient gesundheitliche Ungleichheit verursachen, stellt sich die Frage, ob diese Ungleichheit *ungerecht* ist. Hierzu liegt umfangreiche Literatur vor [31, 35, 36]. Dabei ist relevant, *welche* gesundheitlichen Ungleichheiten ungerecht sind und aus welchen Gründen: *Wenn gesundheitliche Ungleichheiten als ungerecht identifiziert werden, erhöht sich die moralische Verpflichtung, diese zu verringern* [31].

Ungleichheiten, die sich aus informierten, bewussten Entscheidungen („informed consent“) ergeben, sind moralisch eher unproblematisch, sofern alle relevanten Informationen über mögliche Handlungsoptionen und deren Vor- und Nachteile verfügbar sind, verstanden werden und – sofern die Lebensumstände es erlauben – frei getroffene, informierte Entscheidungen auch umgesetzt werden können. Die Möglichkeit, sich frei und informiert zu entscheiden, ist jedoch deutlich ungleich verteilt. Wenn Ungleichheiten vorliegen, die mit populationsbezogenen Vulnerabilitäten zusammenhängen, also z. B. mit struktureller oder epistemischer Diskriminierung (beispielsweise aufgrund von Hautfarbe, Geschlecht, Herkunft oder sexueller Orientierung) oder mit sozial ungerechten strukturellen Bedingungen (z. B. des Arbeitens, des Einkommens oder des Wohnens), ist es problematisch, auf Eigenverantwortung und einen verbesserten Zugang zu Gesundheitsdienstleistungen oder Technologien zu verweisen. Grund ist, dass individuelle Ansätze strukturelle Ungerechtigkeiten kaum verändern können [16]. Solche Ansätze können womöglich sogar zu ungerechtfertigten Schuldzuweisungen führen, wenn davon ausgegangen wird, dass gesundes Verhalten und Krankheitsvermeidung doch lediglich von Zugang zu Gesundheitsversorgung, Information und Technologie – und nun *sogar* den niedrigschwelligen, häufig kostenlosen mobilen Angeboten – abhinge [37].

Es wird also zum einen diskutiert, ob die digital unterstützten Entwicklungen hin zu mehr Eigenverantwortung zu Schuldzuweisungen führen und somit auch zu Verlusten des Solidaritätsgedankens, einem zentralen Pfeiler des deutschen Gesundheitswesens [38]. In Ansätzen sehen wir diese Entwicklung bereits in den Prämienmodellen der Krankenkassen (teilweise mit Einbindung z. B. von Fitness-Apps), die verhaltensbezogene Präventionsmaßnahmen belohnen [39, 40].

Zum anderen wird davon ausgegangen, dass sich Gefühle von Scham und Stigma bei den Menschen einstellen, die es angesichts komplexer psycho-sozialer Belastungen oder angesichts einer benachteiligten Position in der Gesellschaft nicht schaffen *können*, sich gesund zu verhalten [41]. Der Bonus derjenigen, die es schaffen können, sich gesund zu verhalten, kann zum finanziellen oder psychologischen Malus der anderen werden, die es nicht schaffen können [39]. Stigmatisierung und Schuldgefühle werden so zu weiteren gesundheitsschädlichen Belastungen, die gesundheitliche Ungleichheiten vulnerabler Gruppen weiter verstärken können.

Gesundheitliche Ungleichheiten resultieren somit häufig nicht aus individuellen Handlungen oder aus eigener Verantwortung, sondern aus der jeweiligen gesellschaftlichen Position und daraus, wie (un-)gerecht eine Gesellschaft strukturiert ist, wessen Anliegen gehört oder als relevant erachtet werden und wie sehr soziale Diversität und Ungleichheiten in Entscheidungsprozessen berücksichtigt werden [31]. Diese gesundheitlichen Ungleichheiten, die entscheidend durch soziale Ungerechtigkeiten mitbedingt und dadurch ungerecht sind, nennen wir im Folgenden *soziale Gesundheitsungerechtigkeiten*.

## Verringert oder verschärft die Nutzung von mHealth im Bereich T2DM soziale Gesundheitsungerechtigkeiten?

Inwieweit lässt sich zeigen, ob mHealth im Bereich T2DM soziale Gesundheitsungerechtigkeiten verringert oder verschärft? Die gegenwärtig vorliegende empirische Forschungsliteratur zu Auswirkungen von mHealth-Technologien im Bereich T2DM auf gesundheitliche Ungleichheiten ist äußerst begrenzt. Bisher gibt es keine wegweisenden Erkenntnisse darüber, ob Nutzen oder Schaden von mHealth innerhalb von benachteiligten Gruppen überwiegen oder benachteiligte Gruppen weniger oder mehr als sozial besser gestellte Gruppen von mHealth-Technologien profitieren. Grundsätzlich ist diese Frage sehr komplex, und eine eindeutige Antwort ist auch in Zukunft nicht zu erwarten. Allerdings halten wir es für notwendig, der Frage mehr Raum in Forschung und Praxis einzuräumen und sie so weit wie möglich, auch in Zukunft, empirisch und normativ auszuleuchten. In diesem Abschnitt werden wir daher auf Aspekte verweisen, die in der ethischen Diskussion von mHealth noch immer zu wenig berücksichtigt werden. Für die folgende Analyse dieser Frage gehen wir von den oben erarbeiteten zentralen Punkten aus:

1. mHealth soll im Bereich T2DM den Trend hin zu gesundheitlichem *Empowerment* und Eigenverantwortung für Gesundheit fördern,
2. Ausbildung und Verlauf von T2DM werden durch einen sozialen Gradienten mitbestimmt,
3. gesundheitliche Ungleichheiten haben eine ethische Relevanz und müssen auch aus einer gerechtigkeitstheoretischen Perspektive diskutiert werden.

### Verringern mHealth-Technologien soziale Gesundheitsungerechtigkeiten im Bereich T2DM?

Soziale Gesundheitsungerechtigkeiten im Bereich T2DM hängen mit sozialen Determinanten wie Wohnort, Altersgruppe, Arbeitsbedingungen, Rassismuserfahrungen und Einkommen zusammen. Wie oben erwähnt, ist eine zentrale Hoffnung, die mit der Nutzung von mHealth verknüpft wird, die Zugangserleichterung zu Gesundheitsdienstleistungen. Ermöglicht wird diese durch die zunehmende Anzahl von Smartphone-Nutzenden und der niedrigschwelligen Verfügbarkeit von Anwendungen. Studien zeigen, dass Patient*innen in benachteiligten Regionen schlechtere Behandlungsergebnisse erzielen als in wohlhabenderen Regionen [42]. Gerade für vulnerable Bevölkerungsgruppen, die zeitlich und örtlich eingeschränkt sind, ist es somit gut vorstellbar, dass mHealth die Präventions- und Behandlungsmöglichkeiten erleichtert und verbessert [43, 44].

Eine systematische Übersichtsarbeit zum Einsatz von mHealth-Technologien bei Personen mit T2DM aus benachteiligen und vulnerablen Gruppen zeigt tatsächlich Hinweise darauf, dass mHealth die Diabeteskontrolle verbessern sowie die Inanspruchnahme des Gesundheitswesens und gesundheitsbezogene Kosten reduzieren kann. Die Autor*innen betonten jedoch, dass die in der Arbeit evaluierten Studiendesigns inadäquat sind, um herauszuarbeiten, *wie* Technologie Nutzer*innen-zentrierter werden kann, um Zugang und Nutzbarkeit der Interventionen für benachteiligte Gruppen zu verbessern sowie deren Bedürfnisse und Bedarfe besser zu berücksichtigen [24]. Insgesamt ist die Datenlage zu benachteiligten oder vulnerablen Bevölkerungsgruppen schwach. Einzelstudien haben gezeigt, dass unterversorgte Bevölkerungsgruppen mit T2DM ein hohes Maß an Interesse und Bereitschaft zeigen, mHealth-Lösungen zu verwenden [45, 46].

Song und Frier führen an, dass besonders bei Kindern und Jugendlichen die Prävalenz für T2DM zunimmt und vermehrt präventive Maßnahmen benötigt werden [47]. Für die sogenannten *Digital Natives* stellt mHealth dabei eine Möglichkeit dar, dieser Herausforderung zu begegnen.

Einige Studien zeigen, dass ethnische Minderheiten häufiger von T2DM betroffen sind und häufiger kardiovaskuläre Komplikationen entwickeln [48, 49]. Es ist zudem bekannt, dass diese Personengruppen – auch in Interaktionen mit Klinikpersonal – Diskriminierungs- und Stigmatisierungserfahrungen machen. Auch hier könnten mHealth-Technologien zumindest symptomatisch Abhilfe verschaffen. Der digitalisierte Zugang zu Gesundheitsversorgung könnte dazu beitragen, Ängste vor Diskriminierung und Stigmatisierung zu verringern, welche bei persönlichen Interaktionen in Kliniken oder Praxen entstehen könnten [50].

Somit hat mHealth auch im Bereich T2DM das Potenzial, die Teilhabe *aller* zu erleichtern und die Gesundheit insbesondere auch sozial benachteiligter Gruppen zu verbessern sowie soziale Gesundheitsungerechtigkeiten zu reduzieren.

### Verschärfen mHealth-Technologien soziale Gesundheitsungerechtigkeiten im Bereich T2DM?

Die digitale Partizipation innerhalb der Bevölkerung ist ungleich verteilt, was in der Literatur als *Digital Divide* beschrieben wird [51]: Menschen ohne Internetzugang oder Zugang zu digitalen Angeboten werden von den potenziell positiven Versorgungseffekten durch mHealth ausgeschlossen. Anders als oft vermutet, spielt dabei nicht nur das Alter eine entscheidende Rolle, sondern auch Bildung, Einkommen, Geschlecht und Migrationshintergrund [12, 13, 51].

Ein gleichberechtigter Zugang zur Technologie ist notwendig, um soziale Gesundheitsungerechtigkeiten zu reduzieren, jedoch nicht hinreichend. Die Gerechtigkeitsdimension geht über Fragen des Zugangs hinaus. Die umfassenderen Aspekte der Gerechtigkeit kommen in der ethischen Fachliteratur bisher im gesamten Bereich der Digitalisierung und künstlichen Intelligenz (KI), aber auch im Bereich mHealth nur unzureichend zur Sprache [13, 52, 53].

mHealth-Technologien werden oft mit einem engen Verständnis der Nutzenden entwickelt und sind nicht unbedingt auf die Bedürfnisse aller ausgerichtet. Die fehlende Berücksichtigung bestimmter Gruppen während der mHealth-Entwicklung bettet sich ein in eine komplexe Historie ungleicher gesellschaftlicher Machtverhältnisse und struktureller Benachteiligungen, die wir hier nicht annähernd wiedergeben und diskutieren können. Allerdings wird dieses Problem konkret benannt und zunehmend kritisch diskutiert. Aus den Science and Technology Studies (STS) ist bekannt, dass Designer*innen und Entwickler*innen oft unterbewusst eine sogenannte *I‑Methodology* anwenden, sich also bei der Technologieentwicklung primär selbst als Referenz für potentielle Nutzer*innen heranziehen [54]. Beispielsweise werden Prototypen meist erst im Team ausprobiert, bevor es größer angelegte Nutzer*innenstudien gibt. Wenn das Team aber – wie im Bereich digitaler Gesundheitstechnologien noch immer häufig – vorrangig männlich und gut ausgebildet ist, werden Bedürfnisse anderer Personengruppen (z. B. Frauen, LGBTQI-Personen und *People of Colour*) übersehen oder nur in klischeehafter Weise mitbedacht [52, 55]. Damit werden bereits im Designprozess Nutzer*innenmerkmale des Entwicklungsteams eingeschrieben [50, 52, 54]. Studien zeigen, dass Schwarze und ethnische Minderheiten, die besonders negative Erfahrungen in der Gesundheitsversorgung machen, oft nicht mitbedacht werden und Algorithmen einen rassistischen Bias aufweisen können [56–58]. Diese Einschreibungsprozesse in der Technologieentwicklung müssen erkannt und stärker reflektiert werden, sonst besteht die Gefahr, dass sich soziale Gesundheitsungerechtigkeiten noch verschärfen, weil bestimmte Bevölkerungsgruppen nicht im gleichen Maße profitieren können wie der „ideale Prototyp“. Hier gibt es auch erste Ideen zur Gegensteuerung, wie beispielsweise das *Digital Health Social Justice Tool Kit*, konzipiert mit Technologieentwickler*innen als Zielgruppe [12]. Ob gesundheitliche Ungerechtigkeit aufgrund rassistischer Verzerrungen in digitalen Gesundheitstechnologien angesichts solcher Toolkits erkannt und adressiert werden können, muss sich noch erweisen. Für eine gerechte, auf mHealth basierende Gesundheitsversorgung ist es jedoch von entscheidender Bedeutung, dass diese Technologien so konzipiert sind, dass sie soziale Diversität berücksichtigen, in keiner Bevölkerungsgruppe Schaden anrichten und dass sie besondere Anstrengungen unternehmen, um soziale Gesundheitsungerechtigkeiten auszugleichen.

Neben Geschlecht, Schichtzugehörigkeit oder Ethnie spielt auch das Alter eine determinierende Rolle. Doukani (2021) hat beispielsweise aufgeführt, dass bei der mHealth-Entwicklung ältere Menschen zu wenig mitbedacht werden oder als homogene Gruppe gesehen werden, die wenig Interesse an neuer Technologie habe [50]. Bagge-Petersen et al. zeigen, dass im mHealth-Designprozess auch die Bedürfnisse von Kindern nicht unbedingt mitbedacht werden [55].

Durch die Erkenntnisse langjähriger Forschung aus STS und Soziologie von Designprozessen ist bekannt, dass Technologie nie neutral sein kann [52, 54]. Um mögliche Verzerrungen und Stereotypen in der Entwicklung von T2DM-bezogenen mHealth-Technologien zu adressieren, sollten weitere empirische Studien durchgeführt werden. Diese können dazu beitragen, dass mHealth allen Bevölkerungsgruppen nutzt und nicht – wenn auch ungewollt – soziale Gesundheitsungerechtigkeiten verschärft.

Erste Arbeiten weisen außerdem darauf hin, dass Menschen mit prekärem sozialem Hintergrund schlechtere Möglichkeiten zur Integration digitaler Technologien zur Gesundheitsprävention in ihren Alltag haben. Selbst wenn prinzipiell Zugang zur Technologie besteht, können oder wollen manche Personen entsprechende Apps angesichts vieler anderer Verpflichtungen und Belastungen nicht priorisieren [14, 59–61]. Die Betonung von Eigenverantwortung für Gesundheit bei gleichzeitig fehlenden psychischen, sozialen und praktischen Freiheitsgraden – und ohne Sensibilität für die strukturellen (teilweise historisch gewachsenen) Benachteiligungen – kann also zusätzlichen Druck und Belastung bedeuten, was zu einer Verschärfung sozialer Gesundheitsungerechtigkeiten führen kann. Um zu verstehen, wie und ob vulnerable Bevölkerungsgruppen digitale Technologien im Bereich T2DM integrieren und nutzen können bzw. wollen, ist auch hier mehr empirische Forschung, die besonders auf die Erfahrungen von Personen zielt (z. B. qualitative Interviewstudien), wünschenswert.

Kritisch diskutiert wird auch der durch mHealth unterstütze Wandel von der Krankheitsbehandlung als Aufgabe des medizinischen Personals hin zu einem *Do-it-yourself*-Projekt der *Gesundheit*. Gesundheit wird dabei zunehmend als individuelle moralische Verpflichtung verstanden und als andauernde Aufgabe der moralischen Selbsttransformation („ongoing moral self transformation“; [62, S. 172]). Es besteht eine individuelle und allgemeine, unermüdliche Erwartung an den perfektionistisch orientierten, disziplinierten Körper, dessen Gesundheitsstatus durch Technologien unterstützt und überwacht werden kann. [62]. Elitarismus, wie er in Online-Communities in den sozialen Medien und bei *Self-Care*-Bewegungen (beispielsweise der *Quantified-Self*-Community) beobachtet wird, kann jedoch zum Ausschluss benachteiligter Menschen führen. Dies ist bei T2DM besonders kritisch zu reflektieren, da aus der sozialpsychologischen Literatur bekannt ist, dass Peer-to-Peer-Unterstützung signifikant ist für eine T2DM-Therapie und soziale Medien hier mittlerweile eine besonders große Rolle spielen [63]. Die Erforschung solcher sozialen Dynamiken in Peer-Support-Gruppen in den sozialen Medien und ihre potenziellen Auswirkungen auf gesundheitliche Ungleichheit stellt ein wichtiges Forschungsdesiderat dar.

Die Risiken im Bereich des Datenschutzes gehören zu den am meisten diskutierten ethischen Aspekten in Bezug auf mHealth. Auch in diesem Bereich können sich soziale Gesundheitsungerechtigkeiten verschärfen, z. B. wenn kostenlose, dafür aber weniger sichere Apps von sozioökonomisch schlechter gestellten Gruppen genutzt werden oder wenn gesundheitsbezogene Daten an Dritte wie Arbeitgeber*innen und Krankenkassen weitergegeben werden. Dadurch können Nutzende als „nicht gesund“ kategorisiert werden und finanziell und psychisch benachteiligt werden. Insbesondere ökonomisch schlechter gestellte Personen könnten somit – angesichts des bestehenden sozialen Gradienten bei T2DM – besonders unter Druck gesetzt werden [37].

## Fazit

Der zunehmende wirtschaftlich und politisch geförderte Einsatz von mHealth unterstützt einen Wandel in der Gesundheitsversorgung hin zu Partizipation, Eigenverantwortlichkeit für Gesundheit und individuellem *Empowerment* von Nutzenden. Die Auswirkungen auf soziale Gerechtigkeit wurden vor dem Hintergrund dieses Trends bisher nur unzureichend diskutiert. In diese Lücke zielt der vorliegende Beitrag. Der Fokus lag dabei auf mHealth im Bereich T2DM, da es hier bereits viel genutzte mHealth-Technologien gibt, der Krankheitsverlauf ganz besonders vom Selbstmanagement der Patient*innen abhängt, und ein eindeutiger sozialer Gradient bei der Erkrankung vorliegt. Allerdings lassen sich die Überlegungen auch auf andere Bereiche übertragen.

Wir konnten zeigen, dass es nicht genügt, soziale Gesundheitsgerechtigkeit bei der Nutzung von T2DM-mHealth daran festzumachen, ob „digital literacy“ gelingt, der Zugang zur Technologie gesichert oder epidemiologisch eine Verbesserung des HbA1c-Werts erreicht wird. Aus einer gerechtigkeitsfokussierten, Public-Health-ethischen Perspektive ist es notwendig, sehr viel nuancierter zu verstehen, wie vulnerable, marginalisierte oder *vergessene* Bevölkerungsgruppen den Einsatz von T2DM-mHealth erleben, welche *sozialepidemiologischen* Auswirkungen der zunehmende Einsatz von mHealth haben kann, welche gesundheitlichen Ungleichheiten im Bereich T2DM ungerecht sind (und warum), inwieweit die gesundheitliche Eigenverantwortung per App in die Hände der Betroffenen gelegt werden soll und wo die Grenzen der Eigenverantwortung liegen.

Der Einsatz digitaler Technologien kann nur dann auch und gerade bei benachteiligten Bevölkerungsgruppen zu einer Gesundheitsverbesserung führen, wenn die sozialen Determinanten von Gesundheit und die strukturellen Bedingungen so ausgestaltet werden, dass es überhaupt möglich ist, eigenverantwortlich bzw. mit der nötigen Unterstützung einen Nutzen aus den Technologien zu ziehen. Eine solche Ausgestaltung geht selbstverständlich weit über den Technologie- und Gesundheitssektor hinaus, darf in den Debatten zu Apps, *Empowerment* und Ethik aber dennoch nicht fehlen. Hierzu ist es auch wichtig, dass während des Design- und Entwicklungsprozesses soziale Diversität und „health equity“ nicht nur Schlagwörter sind, sondern tatsächlich berücksichtigt werden [64].

Werden diese Dimensionen vernachlässigt, können gesundheitliche Ungleichheiten und Ungerechtigkeiten durch mHealth sogar verschärft werden. Um dem entgegenzuwirken, sind diverse Akteure gefordert, z. B. aus dem Bereich Forschung und Entwicklung von mHealth (z. B. privater und öffentlicher IT-Sektor), oder Entscheidungsträger, die Einsatz und Auswirkungen von mHealth planen und regulieren (z. B. Gesundheitspolitik und Fachgremien). Nutzende sind aufgerufen, sich durch ihr Erfahrungswissen einzubringen. Insbesondere wenn es um marginalisierte Gruppen geht, sind hier auch Repräsentierende (z. B. Nichtregierungsorganisationen, Sozialarbeiter*innen) gefordert, auf mögliche Missstände oder Unzulänglichkeiten hinzuweisen. Sie können ebenso aufzeigen, wo mHealth sinnvoll und nutzbringend eingesetzt werden kann. Die intensive empirische Erhebung und Beschäftigung mit Erkenntnissen aus Medical Information Sciences, Medizin, Public Health, Global Health und (Sozial‑)Epidemiologie sind notwendig. Zudem ist Kenntnis aus dem Bereich STS und zu Theorien der (Gesundheits‑)Gerechtigkeit geboten. Hier gibt es bereits gut ausgearbeitete Analysen und konzeptuelle Ansätze, die helfen, Ungerechtigkeiten zu erkennen und den Blick auf Strukturen, Epistemologie, Intersektionalität, ungleiche Machtverhältnisse und Marginalisierung zu schärfen sowie Eigenverantwortung auf gerechte Weise zu fördern.

Die Berücksichtigung der sozialen Diversität und der sozialen Determinanten von Gesundheit ist ein stetiger Prozess und muss alle Phasen der Entwicklung und des Einsatzes von mHealth durchziehen. Aus einer Public-Health-ethischen Perspektive muss der Einsatz von mHealth außerdem im Kontext der Verhältnisprävention und der sozialen und politischen Bedingungen von Gesundheit und ihren sozialen und epistemischen Ungerechtigkeiten evaluiert werden.

## Footnotes

1 Stand Oktober 2022 gibt es zwei Apps im Bereich Diabetes (T2DM seit 15.04.2022 und Kombination Diabetes mellitus Typ 1 (T1DM)/T2DM/Depression seit 11.12.2021).
